# Is Pyorrhea a Local or Constitutional Disease?

**Published:** 1922-07

**Authors:** Russell W. Bunting

**Affiliations:** Ann Arbor, Mich.


### IS PYORRHEA A LOCAL OR CONSTITUTIONAL DISEASE?

BY RUSSELL W. BUNTING, D.D.SC., ANN ARBOR, MICH.

In a paper entitled "Late Researches on Interstitial Gingivitis," read before the New York State meeting, May, 1921, Dr. E. S. Talbot[1] reviewed his former work on the subject and elaborated certain newer concepts which he believed to be of great importance. He restated his former assertions that pyorrhea is a disease which is local in its manifestations, but is due to both local and constitutional causes; that bacteria and infectious organisms are not primary causes; that the disease is marked by, first, bone absorption; second, inflammation of the gums and fibrous tissue, and third, pus infection. In corroboration of the latter statements he cites the recent work of Fleischmann and Gottlieb, who state that the absorption of the bony alveolus is the primary causal factor. The theme of the paper, however, dwells largely on the constitutional disturbances arising from malnutri-

---

1 TALBOT, E. S.: "Late Researches on Interstitial Gingivitis." DENTAL COSMOS, 1921, p. 795.

tion and their relationship to peridental disease. The author points to the work of Funk, Holst, Frölich, Mellanby, McCollum and others, who have been producing deficiency diseases in animals and who have noted attendant changes in the maxillary bones and loosening of the teeth. He calls attention to the similarity between the initial lesion of pyorrhea and the bone changes associated with scurvy, and draws the conclusion that many forms of peridental disease are in reality mild forms of scurvy resulting from deficiencies of diet. He further quotes Hess, who says that the chief clinical importance of disorders of nutrition is that the tissues become more susceptible to the invasion of bacteria and their products.

Inasmuch as this paper is confined to a consideration of constitutional causes of pyorrhea, the impression is given that the writer considers the local factors to be of secondary importance; that the disease arises solely from a constitutional disturbance; and that the treatment of the disease consists in the correction of the nutritional or metabolic fault. In the discussion which followed the reading of this paper Merritt and Stillman accentuated the importance of the local causes of pyorrhea and stated that the disease might be successfully treated and cured by strictly local measures. In their opinion constitutional factors, if they be present, may be disregarded and local irritations and infectious processes may be regarded as the more important causes of the disease.

### DIVERGENT OPINIONS AND THEORIES ON THE CAUSES OF PYORRHEA

The controversy which followed strongly reminds one of the old debate between the constitutionalists and the localists, which dates back to the very beginning of our knowledge of pyorrhea. In the early days Magitot was the champion of the constitutionalists, while Serran supported the view that pyorrhea arises solely from local causes. The contention between the two schools of thought became so strong that it was decided to submit the question to a commission of scientists to be appointed by the Société de Chirurgie of Paris.[2] This commission investigated the available data on the subject and brought in the verdict that the disease is not due to local causes, but is rather a manifestation of constitutional disturbance.

At the same time John W. Riggs [3] was emphatically denying that peridental disease arises from constitutional causes, claiming that it begins as a local inflammation of the gingival tissues caused by the irritation of calculi and other accretions on the teeth contiguous to or beneath the free margin of the gum. He based his views very largely on his practical clinical observations that, if in the treatment of pyorrheal infections the accretions be completely removed from the teeth, the disease may usually be arrested and permanently controlled. The results which he obtained by local treatment of the disease aroused widespread interest in the method and many practitioners actively engaged in this form of practice.

During the years which followed, G. V. Black [4] described pyorrhea as a specific inflammatory process in the peridental membrane, while W. D. Miller,[5] failing to isolate any constant type of organisms in the lesions, concluded that the disease was produced by a wide group of organisms in varying combinations. The work of Goadby [6] on vaccine therapy in the treatment of pyorrhea opened the way for extensive clinical experimentation with various forms of stock and autogenous vaccines.[7]

---

[2] *Bulletin et Memoirs de la Soc. de Chir.*, Tome vi, p. 411.

[3] Riggs, J. W.: *Pennsylvania Journal of Dental Science*, Vol. iii, p. 99.

[4] Black, G. V.: "*American System of Dentistry*," Vol. v, p. 953; "*Operative Dentistry*," 1908; "*Special Dental Pathology*," 1915.

[5] Miller, W. D.: "Microörganisms of the Human Mouth," 1890, p. 329.

[6] Goadby: *British Medical Journal*, 1905, Vol. ii, p. 562.

[7] Medalia, L. S.: Dental Cosmos, 1913, p. 24; *Ibid.*, 1916, p. 1000.

This was followed by the amœba theory and the emetin treatment,[8] and more recently it is being stated[9] that spirochetæ are the specific cause of pyorrhea for which various spirocheticides are prescribed. On the other hand, Hartzell[10] claims that the initial lesion of pyorrhea is produced by streptococcus and that the only organisms of importance are those of the streptococcal, staphylococcal, and pneumococcal groups.

Hopewell-Smith[11] believes that pyorrhea begins as an atrophy of the alveolar bone and that the presence of calculus is not sufficient to induce the condition. Talbot,[12] who since 1896 has written voluminously on the subject, takes a middle ground in the discussion. He recognizes the importance of certain local causative factors, such as infection, calculi, etc., but he believes the most significant factor in the process is a preliminary degeneration of the alveolar process. He points out that in the evolutionary development of man a marked degeneration of the alveolar process has occurred, with the result that in the present generation these bony structures are lightly built and are prone to degenerative changes under any stress or nutritional disturbance. He states that the initial lesion consists in preliminary degeneration of the alveolar bone, due to senility, intoxications, malnutrition or other constitutional fault. Local irritative factors

---

8 Chiavaro, A.: *Dental Review*, 1914, p. 1122; Barrett, M. T., Dental Cosmos, 1914, p. 948; Bass and Johns: *Dental Summary*, 1914, p. 994; *Journal American Medical Association*, 1915, p. 553; Hartzell, T. B.: *Journal Allied Dental Society*, 1916, p. 7.

9 Reed, G. H.: *Items of Interest*, 1915, p. 241; Wright: Dental Cosmos, 1915, p. 1003; White: Dental Cosmos, 1915, p. 405; Noguchi, H.: *Journal Experimental Medicine*, 1912, Vol. xvi, p. 103; Kritchevsky and Seguin: Dental Cosmos, 1918, p. 781; 1921, p. 888.

10 Hartzell, T. B.: *Dental Items of Interest*, 1919, p. 45.

11 Hopewell-Smith: Dental Cosmos, 1911, p. 397; *ibid.*, 1913, p. 765, and 1918, p. 426.

12 Talbot, E. S.: Dental Cosmos, 1896, pp. 310, 660; *ibid.*, 1905, p. 310; 1909, p. 1147; 1915, p. 485; *Dental Summary*, 1903, pp. 435, 538; *ibid.*, 1917, p. 282; *Items of Interest*, 1906, p. 837; "Interstitial Gingivitis," 1899 and 1913.

then, acting in conjunction with the underlying degenerative changes, may produce the characteristic lesions of the disease.

Roy,[13] in his review of the subject before the Sixth International Dental Congress, places the greatest emphasis on an initial degeneration of the bone—a condition which he terms "precocious senile degeneration." He believes that local trauma and infection enter into the process of peridental destruction, but considers them to be adjuvant causes and secondary to degenerations in the alveolar bone, which are general and systemic in their origin. He asserts that local irritation, such as mechanical stress, infection, and faulty oral hygiene, when acting alone, produce gingivitis but not pyorrhea, unless a precocious senile degeneration of the alveolar border has taken place. On the other hand, he believes that senile degeneration of the alveolar bone without local irritations produces only an atrophy of the peridental tissues, and *if a rigid oral hygiene be maintained no pyorrheal pockets will be formed.*

From this brief résumé of the theories and opinions regarding the nature of peridental disease, it is evident that there is still considerable divergence of thought concerning the underlying and determining factors involved in the process. This is unfortunate, for the question is an exceedingly important one. If pyorrhea be a local disease caused by some local irritation or infective process, then there is hope that we may prevent and cure it by removing the local causative agents. If, on the other hand, it be a local manifestation of a purely constitutional disease, then our only hope for a successful cure lies in the discovery and correction of the constitutional disturbance which has produced it. This uncertainty regarding the underlying causes and the exact nature of peridental disease has led to consider-

13 Roy, Maurice: Dental Cosmos, 1918, p. 659.

able confusion in the mind of the average practitioner as to whether or not it is possible by any means to prevent and successfully control pyorrhea. It is with these practitioners in mind in the hope of reconciling for them some of the apparently conflicting views and of outlining a reasonable course of procedure in the handling of these cases that this paper has been undertaken.

During all the years in which the controversy regarding the cause and nature of pyorrhea has been carried on, a certain group of operators following the original precepts of Riggs have been successfully treating and controlling pyorrhea by local operative procedures. New theories came and went, but these pyorrhea workers continued in the even tenor of their ways to cleanse surgically the affected root surfaces, to remove all irritants and undue stresses, and to establish permanent mouth cleanliness, as a result of which an arrest of the destructive process and a subsequent healing of the lesions was secured in a large percentage of cases. So beneficial have been the results of those operators who have become proficient in this form of treatment, that there is no longer any room for doubt as to the efficacy of these measures to control many forms of pyorrhea.

If constitutional factors are involved in their cases, these operators have considered them only in a general way, bending their greatest efforts toward the perfection of a careful technique of root surgery and the establishment of thorough mouth cleanliness. In a great majority of cases in which the destructive process has not advanced too for an arrest of the disease is effected and a healing of the lesion is brought about, while in the beginning cases, practically without exception, the pyorrheal tendency is checked and the progress of the disease permanently prevented. By reason of the success which has been obtained by these local procedures, many have been led to believe that pyorrhea is wholly a local disease and is entirely independent of systemic conditions. On the other hand, many who hold to the constitutional origin of pyorrhea believe that local measures, if thoroughly performed, are largely effective in preventing and controlling the disease. Indeed, there are few who have made a close study of the problem and have impartially adjudged the results of these operators who do not agree as to the efficacy of this method of treatment. The question then arises as to how the conception of pyorrhea as an expression of systemic or constitutional disease may be reconciled with the fact that it is amenable to local treatment.

#### VITAL RESISTANCE THE DETERMINING ETIOLOGIC FACTOR

In a former treatise on pyorrhea alveolaris,[14] we have pointed out that the nature and course of the disease under consideration is determined, first, by the degree of health and vital resistance which the tissues immediately surrounding the teeth possess and their ability to withstand the injuries and infectious processes to which they may be subjected, and second, the nature and degree of harmful influences which may affect these tissues. When we consider the wide range of injuries (traumatic, chemic and infectious) which occur in the various cases presented, and the varying types of tissue reaction and resistance which are manifested in different individuals, we then may appreciate that pyorrhea is not a uniform or specific affection, but is rather a sympton complex, having many forms and variations, depending on the combination of factors involved in each individual case.

For instance, in certain mouths the wedging of food on the proximal gum tissues or an overhanging proximal filling will stimulate the injured tissues to proliferation and hypertrophy, while in other individuals the same

---

14 Bunting: "American Textbook of Operative Dentistry," M. L. Ward, 1920, p. 527.

form of irritant will produce an atrophy of the tissues, followed by infection and the formation of deep pyorrheal pockets. In the former type, correction of the fault results in a prompt return to normal, but in the latter the recuperative powers of the tissues are decidedly less active. In many instances the strain of traumatic occlusion results in the degeneration of the peridental tissues and pyorrheal infection, but in other cases, especially in younger individuals, malocclusion and traumatism may be very marked, but there is no resultant peridental disease. Furthermore, there are many patients who have a close interlocking occlusion or abnormal occlusal traumatism, which may be present throughout their youth and early adult ages without manifest harm to the supporting tissues; but, suddenly, between the ages of forty and fifty, or simultaneously with some constitutional stress or disease, the peridental tissues degenerate and pyorrhea ensues. In such cases the local traumatism has been present for a long time, but it was not until the resistance of the local tissues had been lowered by age or constitutional fault that the traumatic injury became effective as a cause of pyorrhea.

It is a matter of common observation that in many mouths which have been neglected there may be an abundance of filth, food débris and calculi in contact with the peridental tissues, which gives rise to severe local irritations and inflammation, but pyorrhea does not occur. Marked acute or chronic gingivitis may be present, but on examination it will be found that the normal attachment of the peridental tissues to the teeth is undisturbed and the characteristic pockets of pyorrhea have not been formed. In other mouths mild irritations of the local tissues are followed by an extensive destruction of the peridental tissues and severance of their attachment to the teeth. Deep pyorrheal pockets occur at the site of irritations which might have been borne easily by the tissues of the first type of mouths. As a rule mouths of the first type are found in strong, healthy individuals, while those of the second type are usually associated with constitutions that are naturally weak and debilitated or are suffering from some systemic disturbance. In both instances the local irritations may be the same, but in one the local tissues successfully resist the injury, while in the other they succumb to disease and infection.

It seems reasonable to conclude, therefore, that the determining factor in the process is not the local irritation to which these tissues may be subjected, but rather the type of resistance which the tissues possess by means of which they may withstand injury. Normally, the peridental tissues are highly resistant to infection and to traumatic injuries incident to the mastication of hard foods. In health the mucous membrane and the underlying tissues about the teeth are hard and firm and are not easily ruptured or torn from their positions. In general it may be said, that the degree of resistance which these peridental tissues possess is to be determined by the general resistive powers of the body as a whole and by the local environmental conditions which surround them.

If the general bodily resistance has been lowered by nutritional disturbances or by disease, it follows that every individual tissue will be less resistant to injury and infection, and the peridental tissues are no exception to this rule. In fact, the alveolar and peridental structures seem to be specially affected by changes in the general health in that they may be disturbed by systemic changes which are mild and often obscure in character. Therefore many individuals, because of a lowered bodily resistance, due to old age, anemia, autointoxication, faulty diet and nutrition, gout, arthritis, diabetes, or other systemic disease, have a lowered resistance of their peridental tissues and are thereby susceptible to pyorrhea. (It was toward one of these causes of lowered bodily resistance, namely, diet and nutrition, that the paper under consideration was directed.)

### LOCAL FACTORS

It may also be noted that pyorrhea is invariably associated with some form of local irritant which seems to determine the location and extent of the disease. For instance, in cases in which the lesions are limited to only a few teeth it will usually be found that there is just at that point some accumulation of calculus, an overhanging filling, an open proximal contact, or traumatic occlusion. The remarkable coincidence of these irritations with the location of pyorrhetic lesions, and the fact that following the removal of the irritating agencies the peridental tissues usually show a marked improvement and return to normal, point in no uncertain manner to the fact that local irritations and stresses constitute active causative factors in the inception and progress of the disease. These local irritants may consist of stress or traumatism, such as calculus, food impaction, abnormal occlusion, or operative fault, by which the gingival and peridental tissues are subjected to indignities greater than they are able to bear. When the resistance of these tissues has been lowered by constitutional fault or continued local irritation, the infective organisms of the mouth may pass nature's first line of defense (the normal mucous membrane and gingival tissues) and invade the deeper structures, producing in them the typical lesions of pyorrhea.

In certain forms of pyorrhea, however, no calculi or other forms of local traumatic stresses are evident. In cases of this type there is invariably present a virulent type of infective organism which, by virtue of its own irritative and invasive powers, is able to attack and destroy the peridental tissues when the systemic and local resistances are low. Especially is this true when the end circulation of the gingivæ has been disturbed by circulating systemic poisons, such as mercury, lead, and intestinal intoxications. These produce passive congestion and lower nutrition of the local tissues which result in a lowered tone and resistance of these tissues to specific types of infection. As we have said, a lowering of general and local resistance is essential to the inception of pyorrhea, but it is also equally true that local injury, either traumatic or infectious, or both, is the *sine qua non* to peridental disease. Constitutional disturbances alone may produce osteomalacia and atrophy of the peridental structures, but without local injuries and infection true pyorrhea does not occur.

In every case of pyorrhea, therefore, it appears that two types of causative factors are involved: first, a constitutional or systemic fault, and second, some form of local injury. It follows then that in the prevention and treatment of the disease both forms of causative factors must be considered. As Dr. Talbot points out in his paper, it is extremely important in the treatment of pyorrhea to discover and to correct the underlying systemic faults which are active in the case. Outstanding disturbances, such as malnutrition, scurvy, intoxications, anemia, and many functional disorders may often be detected and relieved by rational therapeutics or diet. In all such cases improvement in the general health will usually be followed by a marked improvement in the health and resistance of the peridental tissues. The elimination of the constitutional faults combined with removal of all local irritations from the teeth and surrounding tissues will in the great majority of cases suffice to stop the pyorrhetic destruction and effect a permanent healing of the lesions, unless the tissue destruction has been too severe to allow their return to normal.

DIETETIC AND NUTRITIONAL FACTORS

However, in many cases of pyorrhea the constitutional factors are not easily discerned, and if they are evident, it may be impossible to correct them. Frequently mild, insidious debilities exist which seriously affect the general and local tissue resistance, but which even the most careful internist fails to discover. These may produce among other disturbances a marked tendency of the peridental tissues toward pyorrheal affection. It is possible that many of these are nutritional faults and scorbutic in type, as Dr. Talbot suggests, and at some time in the future when we shall know more of the science of diet and nutrition many of these obscure constitutional faults may be corrected. At the present time, however, there are many such cases which defy the best efforts of our diagnosticians and which, in spite of all that we may do in the correction of systemic faults, continue to be predisposed toward pyorrhea.

There are also cases in which the constitutional fault is old age, chronic diabetes or nephritis, arthritis, gouty or tubercular diathesis, constipation and intestinal intoxication, acidosis, obesity, or other metabolic faults, which are not amenable to cure. These disturbances may be subacute or chronic, not severe enough to incapacitate the patient, but may produce a marked lowering of tissue resistance, thereby acting as a constitutional cause of pyorrhea which can not be eliminated. In the treatment of all the foregoing types of pyorrhea in which the constitutional faults can not be determined or are incurable, we are confronted with the fact that certain underlying constitutional causative factors are present in the case which we are unable to eliminate. The question then arises, is it possible to treat and successfully control pyorrhea without removing the constitutional causes involved in the case?

At the present time the only basis upon which we may answer this question is that of clinical data and experience. The very beneficial results which are obtained by a large group of operators who specialize in the local treatment of pyorrhea is strong evidence of the efficacy of this method of treatment which should be given serious consideration. These operators remove the food and calculi, they smooth the surfaces of the enamel and exposed root surfaces, they relieve undue occlusal stresses and restore, as far as possible, the normal physiologic function of the teeth, and withal they reduce the infectious process in the mouth and peridental tissues. All this is followed by a régime of increased care of the mouth by the patient, in which the teeth and mucous tissues are thoroughly scrubbed by stiff-bristled brushes. This serves to periodically remove the accumulations of irritating food and calculi and markedly decreases the infectious processes of the mouth. At the same time the friction of the brush on the mucous membranes serves to stimulate an active flow of blood through the gingival capillaries, which increases the nutritive functions and resistance of these tissues. In fact, it has been noted by every observant practitioner that thorough and rational brushing of the teeth and surrounding mucous membranes alone will suffice to increase the tone and resistance of these tissues to traumatic injury and infection.

### CONCLUSIONS

With these facts in mind it seems reasonable to conclude that pyorrhea is the result of a combination of constitutional and local influences which militate against the health and vital resistance of the peridental tissues. If both general and local causes can be eliminated the disease is checked, and if not too far advanced may be permanently cured. In cases in which the constitutional causes can not be remedied there is sound reason to believe that removal of all local disturbing factors and stimulation of the gingival circulation and local resistive powers will very often check the disease. In this manner the balance of power may be thrown over on the side of tissue resistance even in the presence of pronounced systemic fault. If the tissues be kept continuously free from the return of the local irritants and if the gingival circulation be frequently stimulated by vigorous brushing the disease will be permanently controlled and will not recur.

What has been said concerning the treatment of pyorrhea by either systemic or local measures applies more definitely to cases of incipient pyorrhea and those in which severe tissue involvement has not occurred. It is true that by skilful technique many cases of advanced pyorrhea have been checked and permanently controlled. But this is a difficult and not always successful procedure, which is far less satisfactory than the treatment of these affections in their incipiency before serious damage has been accomplished, at which time they may easily be controlled. If we admit that pyorrhea can not occur without some form of local irritation acting in conjunction with a systemic fault, then we must grant that if the mouth be kept free from local irritant there will be no pyorrhea, even though there be a pronounced constitutional tendency toward the disease. If pyorrhea be discovered in the early stages and if strict oral hygienic measures be promptly instituted and continued, the disease, as a rule may promptly be checked and its progress permanently prevented. Clinical experience has taught us that pyorrhea is the most definitely preventable of all oral diseases and seldom would occur if the mouth were kept free from continued irritation.

In conclusion it may be stated that the writer agrees with Dr. Talbot when he says that pyorrhea is a constitutional disease in that it is often associated with nutritional fault and systemic intoxication or disturbance, and he also agrees with those who hold that it is caused by local injury and infection. That is, that careful diagnosis will reveal both local and systemic factors active in every case. However, of the two types of causes, the local factors are much more easily determined and removed than are the systemic faults. In the great majority of cases of incipient pyorrhea and those in which the disease has not progressed to a hopeless stage, the thorough removal of all irritants and stresses from the teeth and contiguous tissues will stop the progress of the disease even in the presence of a constitutional susceptibility. This involves the removal of all calculi, overhanging fillings, improperly fitted crowns and bridges, food débris and bacterial plaques, the relief of all pronounced occlusal stresses, and the restoration of the teeth to normal physiologic function. The patient must then be taught to keep the mouth clean and to increase the local tone and resistance of the peridental tissues by vigorous massage. If cases of pyorrhea are once placed under control in this manner and occasional assistance be given the patient to keep his mouth free from the recurrence of irritation, the disease may be permanently controlled and, as a rule, will not return.

In our study of pyorrhea in the college clinic during the past several years the principles stated have clearly been borne out by practical experience. Here a large number of cases have been treated by students who, with some assistance, have been able to effectually control the disease and permanently eradicate it in a very large percentage of favorable cases. Many of these cases return after several years, during which they have had no attention and, as a rule, if the patient has carried out instructions, the mouth is in good condition and little or no evidence of pyorrhea can be found. This is the work of students in a dental clinic. What students can do any practitioner can do. Pyorrhea is a definitely preventable disease which should seldom if ever occur in mouths of patients under regular supervision. When it has occurred the great majority of cases, when taken in time, may be permanently controlled. The question is, shall we do it?

